# Long Tree-Ring Chronologies Provide Evidence of Recent Tree Growth Decrease in a Central African Tropical Forest

**DOI:** 10.1371/journal.pone.0120962

**Published:** 2015-03-25

**Authors:** Giovanna Battipaglia, Enrica Zalloni, Simona Castaldi, Fabio Marzaioli, Roberto Cazzolla- Gatti, Bruno Lasserre, Roberto Tognetti, Marco Marchetti, Riccardo Valentini

**Affiliations:** 1 Department of Environmental, Biological and Pharmaceutical Sciences and Technologies, Second University of Naples, Caserta, Italy; 2 Ecole Pratique des Hautes Etudes, Centre for Bio-Archaeology and Ecology, Institut de Botanique, University of Montpellier 2, Montpellier, France; 3 Department of Mathematics and Physics, Second University of Naples, Caserta Italy; 4 Department for Innovation in Biological, Agro-food and Forest Systems. Università della Tuscia (DIBAF), Viterbo, Italy; 5 Department of Biosciences and Territory, University of Molise, Pesche, Italy; 6 The EFI Project Centre on Mountain Forests (MOUNTFOR), San Michele all'Adige, Italy; University of California Davis, UNITED STATES

## Abstract

It is still unclear whether the exponential rise of atmospheric CO_2_ concentration has produced a fertilization effect on tropical forests, thus incrementing their growth rate, in the last two centuries. As many factors affect tree growth patterns, short -term studies might be influenced by the confounding effect of several interacting environmental variables on plant growth. Long-term analyses of tree growth can elucidate long-term trends of plant growth response to dominant drivers. The study of annual rings, applied to long tree-ring chronologies in tropical forest trees enables such analysis. Long-term tree-ring chronologies of three widespread African species were measured in Central Africa to analyze the growth of trees over the last two centuries. Growth trends were correlated to changes in global atmospheric CO_2_ concentration and local variations in the main climatic drivers, temperature and rainfall. Our results provided no evidence for a fertilization effect of CO_2_ on tree growth. On the contrary, an overall growth decline was observed for all three species in the last century, which appears to be significantly correlated to the increase in local temperature. These findings provide additional support to the global observations of a slowing down of C sequestration in the trunks of forest trees in recent decades. Data indicate that the CO_2_ increase alone has not been sufficient to obtain a tree growth increase in tropical trees. The effect of other changing environmental factors, like temperature, may have overridden the fertilization effect of CO_2_.

## Introduction

Africa's forest cover is estimated at 650 million ha, constituting 17% of the world's forests [1]. Because African forests are so rich in carbon, their net loss or uptake of carbon has important implications for atmospheric CO_2_ levels [2].

A continued rise in the atmospheric CO_2_ concentration, increased nutrient deposition and climatic changes are likely to have affected tree growth and forest dynamics [3]. Higher CO_2_ concentrations may stimulate plant growth directly through enhanced photosynthesis [4, 5], and indirectly through reduced water consumption by plants and hence slower soil moisture depletion. Recent studies have reported that growth rates of tropical forests have accelerated over the past few decades [6, 7] in agreement with the hypothesis of increased productivity caused by rising concentrations of atmospheric CO_2_ and carbon fertilization [8, 9]. However, at present, our state of knowledge about the long-term effects of CO_2_ on tropical tree growth is largely underpinned by experimental evidences collected from CO_2_ enrichment experiments and paleo-records for which little process attribution is possible. Most studies to ascertain the effects of anthropogenic climate change on tropical forests, deal with seedlings in growth chambers [9, 10, 11] or with mature trees under controlled conditions (Free Air CO2 Enrichment experiments—FACE) [12, 13, 14]. Results from these studies are limited by their restriction to small trees, artificial growing conditions with high availability of water and nutrients and growth in the absence of plant competition and of other natural stressors, or simply by the unnatural and fast induced increases in CO_2_ [15].

On the other hand, studies on tropical forests have focused primarily on changes in stand-level growth rates (i.e. change in total basal area or biomass per unit area). The most widely used form of monitoring tree biomass accumulation is that of permanent plots [13, 6, 7, 16], with dendrometric measurements (i.e. diameters and heights with a frequency of two or five years). In this case, limitations come from the generally short duration of these studies (of the order of decades), due to the relatively recent establishment of the plots, with the result that many long-term patterns of tropical tree growth have been extrapolated using short-term data.

Zuidema et al. (2013) [17], in their attempt to fill knowledge gaps about African forests and global change, highlighted the need for a long-term trend approach, at a scale at least of centuries. In this way the real lifespan of forests could be considered, i.e. the time period required to acclimate to atmospheric changes [18]. Indeed, with short-term experiments it is not possible to ascertain whether trees adjust their physiological response to the gradual increase of CO_2_ concentrations, and how quickly they implement it [19]. Several studies attest a decline in the CO_2_ fertilization effect with tree age: the effect of the early growth stimulation appears to become less important after a few years in mature trees [13, 20, 21, 22]. Further, there is poor understanding of the interactive effects of CO_2_ enrichment, current increased temperature, and changing rainfall on tree populations and tropical forest communities [17]. Therefore, a long time interval analysis of trend patterns in future studies is critical in assessing the long-term response of trees to environmental factors in order to avoid biased results.

Tree rings are good proxies of climate information: they offer insight into lifetime growth patterns, allowing climate impacts on trees to be evaluated [23]. Furthermore, tree rings are considered as a significant indicator of CO_2_ fertilization, because woody stems are the most permanent biomass carbon pool in vegetation and ring widths reflect carbon storage dynamics [24]. In particular tree-ring width, converted into basal area increment (BAI), is a reliable proxy for total carbon uptake [25], because it represents the overall woody growth of the tree as a two-dimensional measure rather than a linear one, such as stem diameter or ring width [26].

In comparison to the large number of papers related to boreal and temperate forests there have been few dendrochronological studies of tropical trees. This is linked to the difficulty of measuring tree rings in several tropical species where trees have an almost continuous growth that prevents the formation of annual rings. However, it has been demonstrated that a large number of species presents annual tree rings [27] and that ring boundary formation is induced by drought or by periodic inundations [27, 28, 29].

Despite existing knowledge, tree ring research has been conducted much more in tropical forests of Latin America and Asia than those in the Africa. Moreover, tree ring chronologies of African species have been developed mostly for semi-arid savannas and miombo woodlands in Southern Africa [30, 31, 32, 33, 34, 35] and especially for the dry tropics in Ethiopia [36, 37, 38, 39]. Limited data are available for West and Central African forests [29, 36, 40, 41], while a recent article [42] linked tree growth and plant water use efficiency to the recent exponential increase of atmospheric CO_2_ concentration.

We used tree rings to ascertain current and past tree growth of tropical trees in the central region of Africa in order to address the following questions: Can we reconstruct lifetime growth patterns of tropical trees in Central Africa’s forests? Do these trees respond to climate driving forces? Has their growth been stimulating by increasing CO_2_ concentration and have they been accumulating biomass over time, in the form of BAI? This information would allow us to make inferences on the magnitude of carbon uptake in Central African forests.

We analyzed three widespread tropical species from the equatorial rain forests of Cameroon to compare biomass accumulation that took place under pre-industrial levels of CO_2_ atmospheric concentration (before the 1850s), with that occurring during the twentieth century. We hypothesized that: 1) there has been a change in species-specific tree growth during the last two centuries; 2) this change is attributable to anthropogenic climate change; 3) inter-annual variability in seasonal patterns of rainfall and temperature is related to the rate of tree growth.

## Materials and Methods

### Ethics Statement

All sampling sites were located on private lands of “Société d’Exploitation Forestière et Agricole du Cameroun (SEFAC)”. The necessary permits for field sampling were issued by the SEFAC authorities. The locations were not protected areas, and the field studies did not involve endangered or protected species.

### Study area

**Fig 1:**
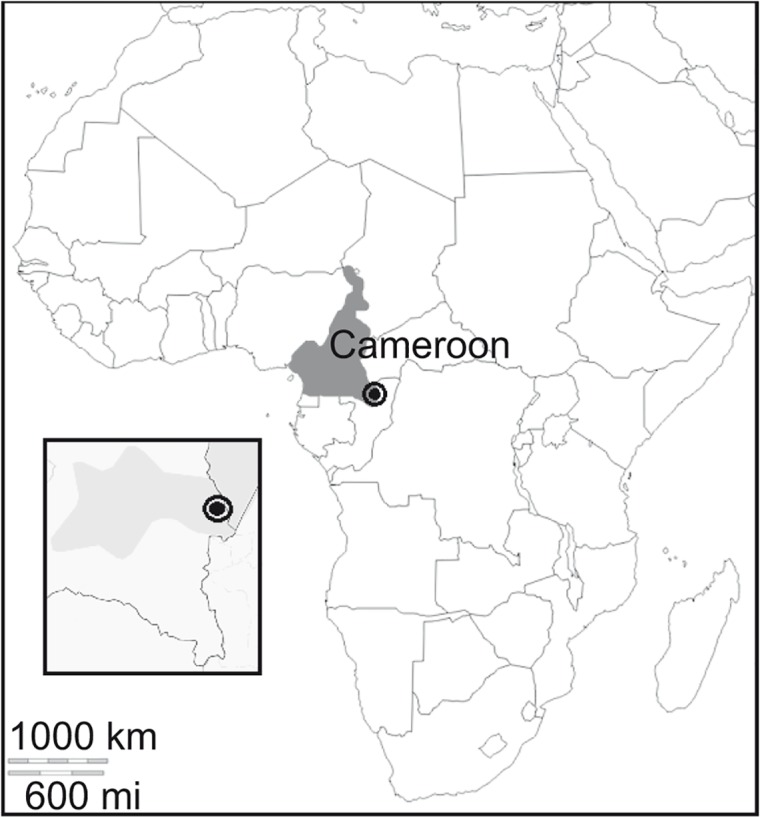
Sampling area. Sampling area (black circle) (2°14ʹ59˝N and 16°9ʹ59˝E), located in the Lobeke National Park, in south-eastern of Cameroon (grey area), within the Congo basin.

The sampling area is Libongo (2°14ʹ59˝N and 16°9ʹ59˝E), located in the proximity of the Lobeke National Park, in south-eastern of Cameroon, within the Congo basin (Fig. 1). It is bounded on the east by the Sangha River, which serves as Cameroon’s international border with the Central African Republic and the Republic of the Congo. The area is covered by a semi-evergreen forest of 2178.54 km^2^, which ranges from 300 to 750 m in altitude above sea level. The soil is a red or yellowish-red oxisol with a high content of clay and oxides of iron and aluminum. The average annual rainfall is 1400 mm, with the dry season occurring from December through February, and an annual average temperature of 24°C. The annual average air humidity varies between 60 and 90% [43].

### Tree species description

The studied species are: *Entandrophragma cylindricum* Sprague (commonly known as Sapele), *Triplochiton scleroxylon* K. Schum. (Ayous) and *Erythrophloeum ivorense* A.Chev. (Tali).

The Sapele (Family Meliaceae) is a widespread species, ranging longitudinally from Sierra Leone to Uganda, and latitudinally from DR Congo to Cabinda (Angola). It is a non-pioneer light-demanding species, with low growth rates under natural conditions, although it was indicated as exceptionally shade-tolerant after studies in DR Congo (PROTA Database, http://database.prota.org/). It is a deciduous tree up to 55–65 m tall, bole up to 200–280 cm in diameter, with low, blunt buttresses up to 2 m high, rarely up to 4 m. Natural regeneration is often scarce in natural forest, but logging operations creating gaps may promote its regeneration. *Entandrophragma cylindricum* (*E*. *cylindricum*, hereafter) provides one of the commercially most important timbers of Africa in terms of quantities produced as well as wood quality.

The Ayous (Family Sterculiaceae) is widely distributed in the West and Central African forests from Guinea east to the Central African Republic, and south to Gabon and DR Congo. It is a light-demanding pioneer species with high growth rates. It is a large deciduous tree up to 50 m tall, reaching 150–210 cm in diameter, with low to very high (up to 8 m) buttresses. Seedlings may be very abundant in large forest gaps, and this species is characteristic of secondary forests. *Triplochiton scleroxylon* (*T*. *scleroxylon*, hereafter) is the major timber tree of West and Central Africa; it is economically the most important timber species of Ghana and Cameroon, making up to 70% of the volume of timber products exported from Ghana and 35% from Cameroon (PROTA Database, http://database.prota.org/).

The Tali (Family Caesalpiniaceae) occurs from Gambia to the Central African Republic and Gabon. It is a non-pioneer light-demander tree up to 40 m tall. Seedlings are often found in smaller forest gaps. *Erythrophleum ivorense* (*E*. *ivorense*, hereafter) has recently gained importance as a timber tree, especially in Cameroon (PROTA Database, http://database.prota.org/).

### Tree sampling and ring-width chronologies

Sampling was carried out from March to May 2011 over a total area of 75 hectares. Trees, ranging in size from 5 to >100 cm diameter at breast height (dbh) were randomly sampled, covering the whole local dbh distribution (mean dbh 50 ± 29 cm). Partial cross sections were collected from individual trees of Sapele, Tali and Ayous specimens. Twenty-five samples of Sapele, 25 of Ayous and 15 of Tali were sampled for this study. All the discs were air-dried and sanded to a high degree using progressively a finer texture of sand paper, from 80- to 600-grit. Preliminary research of bibliographic information about the anatomic features of the studied species [44, 45, 46] allowed us to identify tree ring widths correctly. Well-sanded samples were first observed under the stereomicroscope (Olympus BH-2, Hamburg, Germany). Two radii per cross section were measured to the nearest 0.01 mm using LINTAB measurement equipment (Frank Rinn, Heidelberg, Germany), coupled to a stereomicroscope and analyzed with TSAP software (Frank Rinn, Heidelberg, Germany).

TSAP software first allowed the series to be visually cross-dated so as to identify missing rings, common marker years and ring width patterns [47]; and then raw ring width chronologies of each dated tree were statistically correlated, according to the Gleichläufigkeit (GLK- a measure of the year-to-year agreement between the interval trend of two chronologies based upon the sign of agreement) and the Crossdate Index (CDI) values. The program COFECHA [48] was run to validate the cross-dating and measurements, and to find potential errors. Once all measurement series had been validated, tree-ring chronologies were developed. ARSTAN software [49] was used to standardize individual chronologies for each species. Sapele and Ayous ring-width series were detrended with a 50-year cubic smoothing spline function, the shorter Tali series with 20-years function. Each choice was made in order to limit the most of the autocorrelation and to emphasize higher inter-annual frequency variations. Cubic smoothing spline curves are efficient to remove non-climatic noise, such as long-term trends and effects of localized disturbance events that characterize natural forest dynamics; at the same time they can cause the removal of possible low-frequency climatic information [50]. For each species, b-weight robust mean was used to develop a mean standardized chronology (STD). Several descriptive statistics commonly used in dendrochronology were used to compare species chronologies. These included standard deviation (SD), which estimates the variability of measurements for the whole series; mean sensitivity (MS), which is an indicator of the mean relative change between consecutive ring widths and is calculated as the absolute difference between consecutive indices divided by their mean value; mean RBAR, which is a measure of the common variance between the single series in a chronology and series intercorrelation (SI), which is a measure of the strength of the signal (typically the climate signal) common to all sampled trees at the site.

The expressed population signal (EPS) was calculated within each site to indicate the level of coherence of the constructed chronology and how it portrays the hypothetical perfect population chronology. Running RBAR and running EPS were computed using a 50-year moving window with a 25-year overlap for *E*. *cylindricum* and *T*. *scleroxylon*, and a 20-year moving window with a 10-year overlap for *E*. *ivorense*. Their values illustrated changes in the strength of common patterns of tree growth over time (S1 Fig.).

To provide a measurement of the change in surface area of each increment around the entire trunk, we calculated the annual basal area increment (BAI) for the period 1800–2000 (from 1900 to 2000 for the youngest Tali):

$$BAI_{n}=\pi r_{n}^{2}-\pi r_{n+1}^{2}$$

where, n is the number of tree rings (increasing towards the tree center) from the outermost whole ring (where n = 1), r_n_ is the radius at breast height (1.3 m) minus the radial increment contributed by bark and the current year partial ring formation at n = 1, and r_n+1_ is the radius at increment n+1, calculated by subtracting the width of the n increment from r_n_.

BAI was used instead of ring width directly, because BAI is less dependent on age and thus avoids the need for de-trending [51]. However, in our analyses of growth trends over time, in order to account for the confounding ontogenetic effects existing in BAI series, we performed a sensitivity analysis (S1 Information and S2–S7 Figs.), by applying different standardization methods. BAI curves were detrended with a 50-year cubic smoothing spline function, while the tree-ring width chronologies were detrended with the regional curve standardization (RCS) technique [52] and the final results were compared to raw data.

### CO_2_ and climate data

The CO_2_ records used for this study were derived from the ice cores obtained at Law Dome, East Antarctica, one of the sites with the highest rate of carbon accumulation (http://cdiac.ornl.gov/trends/co2/lawdome.html). Since Law Dome dataset does not allow us to detect the annual concentration, spline fitting [53] and interpolation for dataset extension to annual resolution of temperature anomalies and mean precipitation for the period 1800–2000 were derived from the Berkeley Tavg and the CRU TS 3.10.01 gridded datasets, respectively, with 1.0° spatial resolution (reference grid 16–16.1E, 2–2.1N). Indeed, local weather stations can provide fragmented and non-homogenized data, which barely cover the twentieth century. The only dataset that extends the African climate record back to the nineteenth century is a semi-quantitative rainfall pattern reconstruction, which combines recent data from more than 300 weather stations, with historical information such as lake level chronologies, landscape descriptions, archives and diaries with references to famine, drought and agriculture [54, 55, 56, 57]. This dataset was compared with the continuous climate time series reported in the KNMI Climate Explorer website (http://climexp.knmi.nl) to verify their reliability.

### Data analyses

The BAI of each species and CO_2_ concentration data were summed and grouped into 50-year (for *E*. *cylindricum* and *T*. *scleroxylon*) or 20-year intervals (for *E*. *ivorense*) to allow a better comparison of trends on the long-term scale. Cumulative average basal area over 50-year intervals for *E*. *cylindricum* and *T*. *scleroxylon* and 20 for *E*. *ivorense* were used to assess the statistical significance of the measured growth rates. In details, i) individual radius measurements for each species were first averaged and their dispersion estimated using the error of the mean; ii) average basal area was estimated by average radii and their uncertainties; iii) cumulative average basal area was determined by summing the average basal area and propagating uncertainties; iv) the datasets produced were plotted against the average year in order to proceed to a linear weighted fitting statistical procedure; v) significance of the observed slope (i.e. growth rate) was assessed by a t test. Fitting procedure was performed by the χ^2^ method. Linear fitting parameters (i.e. slope and intercept) uncertainties were estimated by the applied procedure when observed χ^2^ was in agreement with the number of degrees of freedom (DGF) of the analyzed system, while fitting procedure was handled to account for deviations of the data points from the linear model when observed χ^2^ was statistically different from system DGF. Measured slopes were t-tested versus zero (i.e. no slope) by means of their uncertainties to assess their consistency.

Multiple regression models and analyses of variance (ANOVAs) were used to identify significant correlations between BAI (dependent variable) and CO_2_, temperature, precipitation and their interaction (independent variables) from 1900 onward, when climate data started to be available and the CO_2_ level increase rapidly. We performed all statistical analyses using the SPSS statistical package (SPSS Inc., Chicago, IL, USA).

## Results

### Tree rings formation and distinctness of ring boundaries

**Fig 2:**
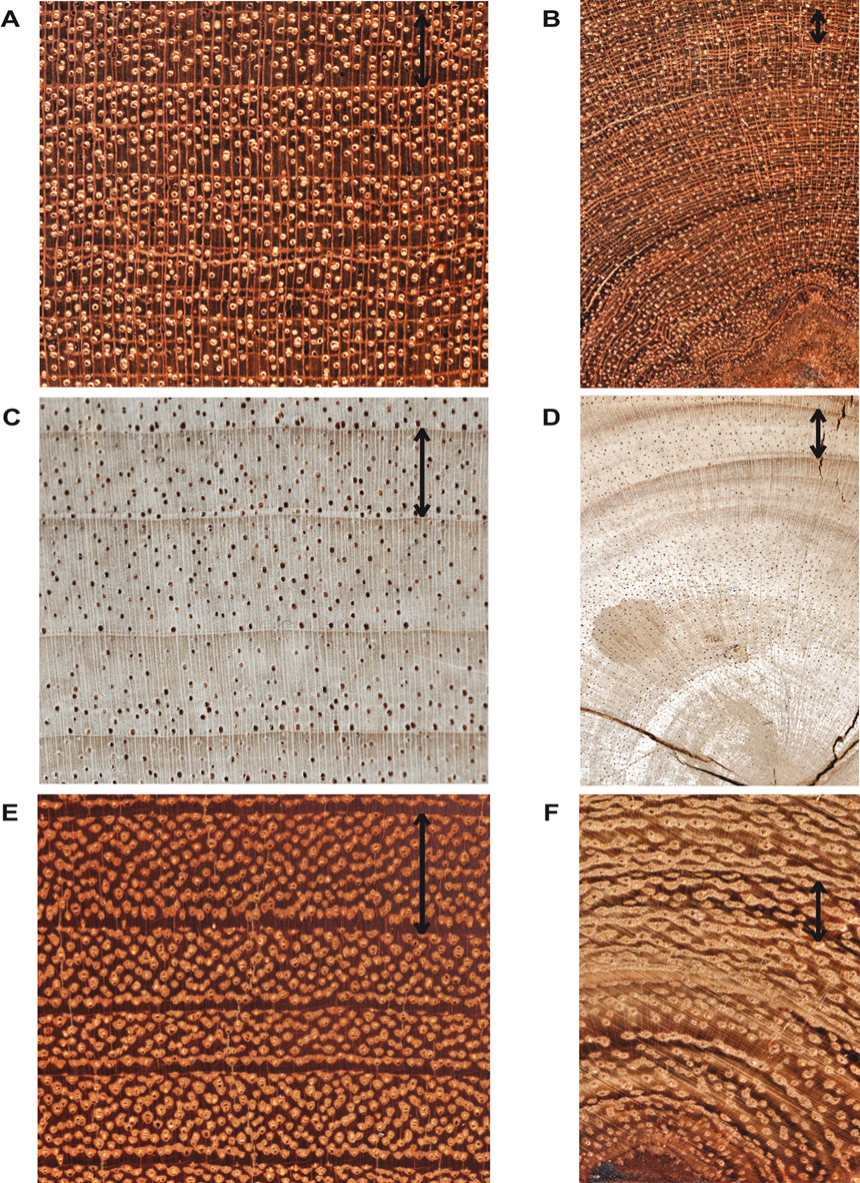
Light microscopy views of cross-sections of the three species. Light microscopy views of cross sections of old (a) and juvenile rings (b) of *E*. *cylindricum*, of old (c) and juvenile rings (d) of *T*. *scleroxylon* and of old (e) and juvenile rings (f) of *E*. *ivorense*. Arrows indicate a whole tree ring.

The distinctiveness of the ring growth varied among the species and was related to species-specific features of wood anatomy. Growth boundaries of the rings of all three species were characterized by thin parenchyma bands, which were difficult to identify for different reasons. *E*. *cylindricum* (Fig. 2a) was characterized by narrow rings rich in axial parenchyma. *E*. *ivorense* (Fig. 2c) showed extremely light rings. Only for *T*. *scleroxylon*, the identification of tree rings was relatively simple, as rings were larger than in the other species and they showed a visible difference in the wood density between earlywood and latewood (Fig. 2b), although growth boundaries were barely perceptible. All three species presented some intra-annual density fluctuation, though they were not considered problematic for dating, since they were not present on the whole tree cross section. The two most difficult areas to identify concerned the juvenile rings (<10 years), where the ring boundaries were almost absent in the region closest to the pith (Fig. 2c, d, e), and the last period of life of the trees, which is located in the outer 10–15 cm of the stem, due to the thinness of its rings, especially for the oldest individuals of *E*. *cylindricum* and *T*. *scleroxylon*.

Further, irregular growth patterns due to knots were found in some samples of *E*. *cylindricum*, while almost all the individuals of *T*. *scleroxylon* showed a bluish discoloration in the sapwood due to the attack of chromogenic fungi.

### Growth patterns in the three species

Cross-dating was successful for all the species with the EPS value >0.85, considered as the minimum value to obtain a sufficiently replicated chronology [58]. Further, Gleichläufigkeit values for each series cross-dated with the mean, range from a minimum of 59 to a maximum of 77 for *E*. *cylindricum*, from 60 to 87 for *T*. *scleroxylon* and from 66 to 94 for *E*. *ivorense*.

**Fig 3:**
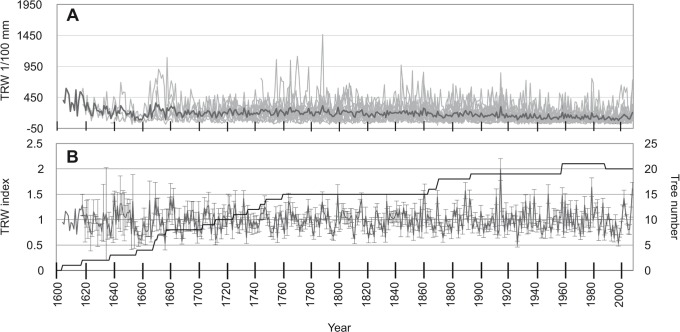
Ring width chronologies of *E*. *cylindricum*. (a) Ring width chronologies of *E*. *cylindricum* with mean chronology (black line) using raw data of average increment rates per tree. (b) Average ring width chronology (n = 25) with standard deviation (SD) of *E*. *cylindricum* after detrending. The number of samples is reported.

**Fig 4:**
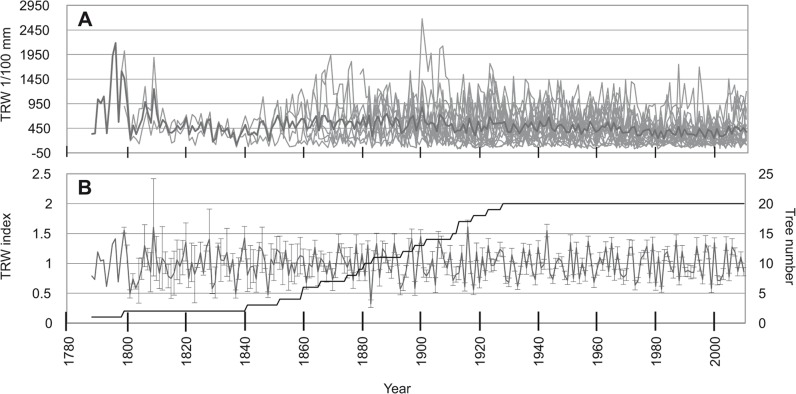
Ring width chronologies of *T*. *scleroxylon*. (a) Ring width chronologies of *T*. *scleroxylon* with mean chronology (black line) using raw data of average increment rates per tree. (b) Average ring width chronology (n = 25) with standard deviation (SD) of *T*. *scleroxylon* after detrending. The number of samples is reported.

**Fig 5:**
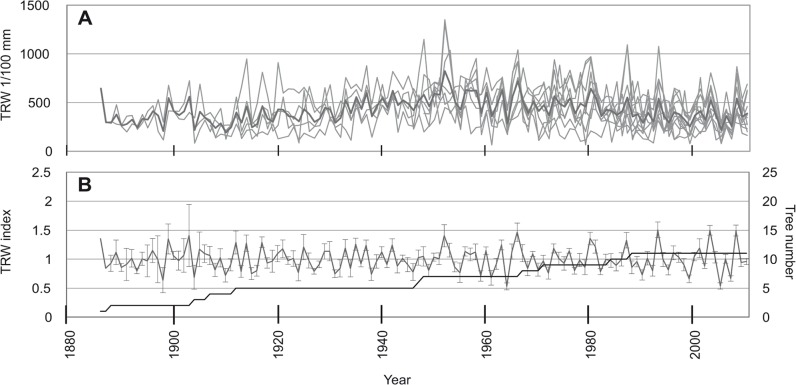
Ring width chronologies of *E*. *ivorense*. (a) Ring width chronologies of *E*. *ivorense* with mean chronology (black line) using raw data of average increment rates per tree. (b) Average ring width chronology (n = 15) with standard deviation (SD) of *E*. *ivorense* after detrending. The number of samples is reported.

The longest mean chronology was that of *E*. *cylindricum* (Fig. 3) with a length of 406 years, followed by *T*. *scleroxylon* with a length of 222 years (Fig. 4) while the shortest was recorded for *E*. *ivorense* (Fig. 5), with 124 years. The standard error ranged from 0.01 to 0.91 for *E*. *cylindricum* standard chronology (Fig. 3b), from 0.02 to 0.81 for *T*. *scleroxylon* (Fig. 4b) and from 0.04 to 0.54 for *E*. *ivorense* (Fig. 5b). The error was visibly higher for the left half of all the curves, because of the scarcity of samples covering the years 1600–1800 for *E*. *cylindricum*, 1800–1900 for *T*. *scleroxylon*, and for about the first twenty years for *E*. *ivorense*.

Mean ring widths of the entire raw chronologies and relative to the period 1800–2000 are reported in Table 1. *T*. *scleroxylon* showed the overall highest mean radial increment (≈ 5 mm) while the lowest one was recorded for *E*. *cylindricum* (≈ 1 mm). All further statistical analyses were run for the period 1800–2000, with the aim of highlighting growth changes from the beginning of the Industrial Revolution to the present. Descriptive statistics of the mean STD chronology of each species for the period 1800–2000 are also listed in Table 1. Standard deviations of *E*. *cylindricum*, *T*. *scleroxylon* and *E*. *ivorense* were ±0.19, ±0.23 and ±0.18, respectively; mean sensitivity (MS) showed values of 0.22, 0.32 and 0.24, respectively; while mean series intercorrelation had values of 0.70, 0.61 and 0.58, respectively.

**Table 1:** Descriptive statistics for raw and standard chronologies of the three species.

	*Entandrophragma cylindricum*	*Triplochiton scleroxylon*	*Erythrophleum ivorense*
Sampled trees (n#)	25	25	15
Raw MRWtot (mm)	1.92	5.13	4.21
Raw MRW1800–2000 (mm)	1.63	4.85	4.35
SD	0.19	0.23	0.18
MS	0.22	0.32	0.24
Glk	59–77	60–87	66–94
SI	0.70	0.61	0.58
RBAR	0.62	0.60	0.57
EPS	0.89	0.86	0.85

Descriptive statistics for raw and standard chronologies of *E*. *cylindricum*, *T*. *scleroxylon* and *E*. *ivorense*. MRWtot = Mean Ring Width of the entire raw mean chronology; MRW1800–2000 = Mean Ring Width of raw mean chronology relative to the period from 1800 to 2000; SD = Standard Deviation; MS = Mean Sensitivity; Glk = Gleichläufigkeit; SI = series intercorrelation; RBAR = mean r bar; EPS = Expressed Population Signal.

### Trends in atmospheric CO_2_ and climate changes vs tree growth

**Fig 6:**
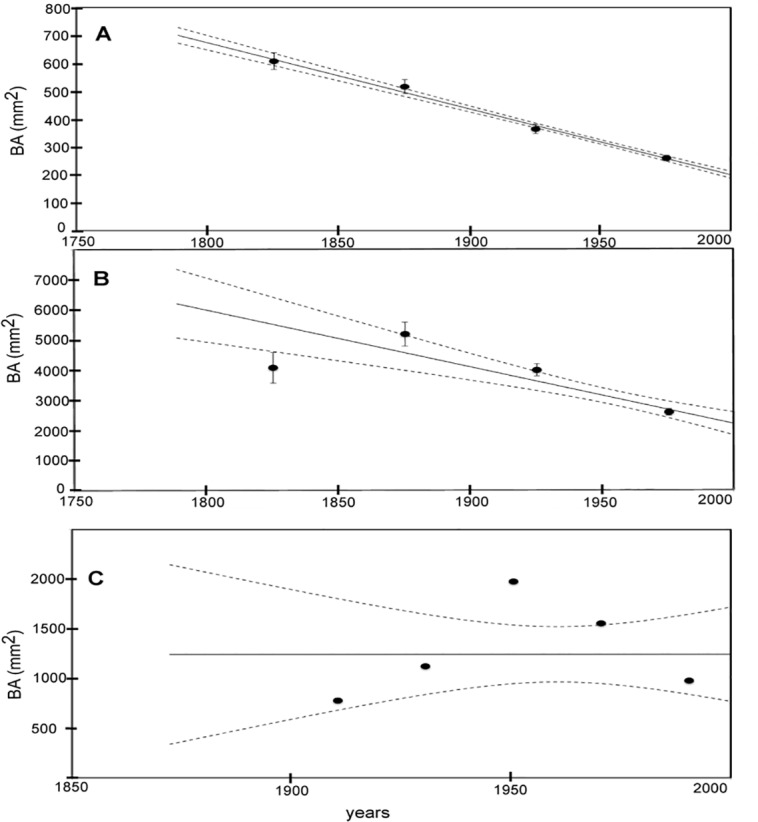
Cumulative average basal area for the three species. Full symbols represent the 50- year cumulative average basal area of *E*. *cylindricum* (a) and *T*. *scleroxylon* (b) and 20 years for *E*. *ivorense* (c). The continuous line represents the weighted best fit linear interpolation with the dashed lines defining the confidence interval of the fit (see text for details)

**Fig 7:**
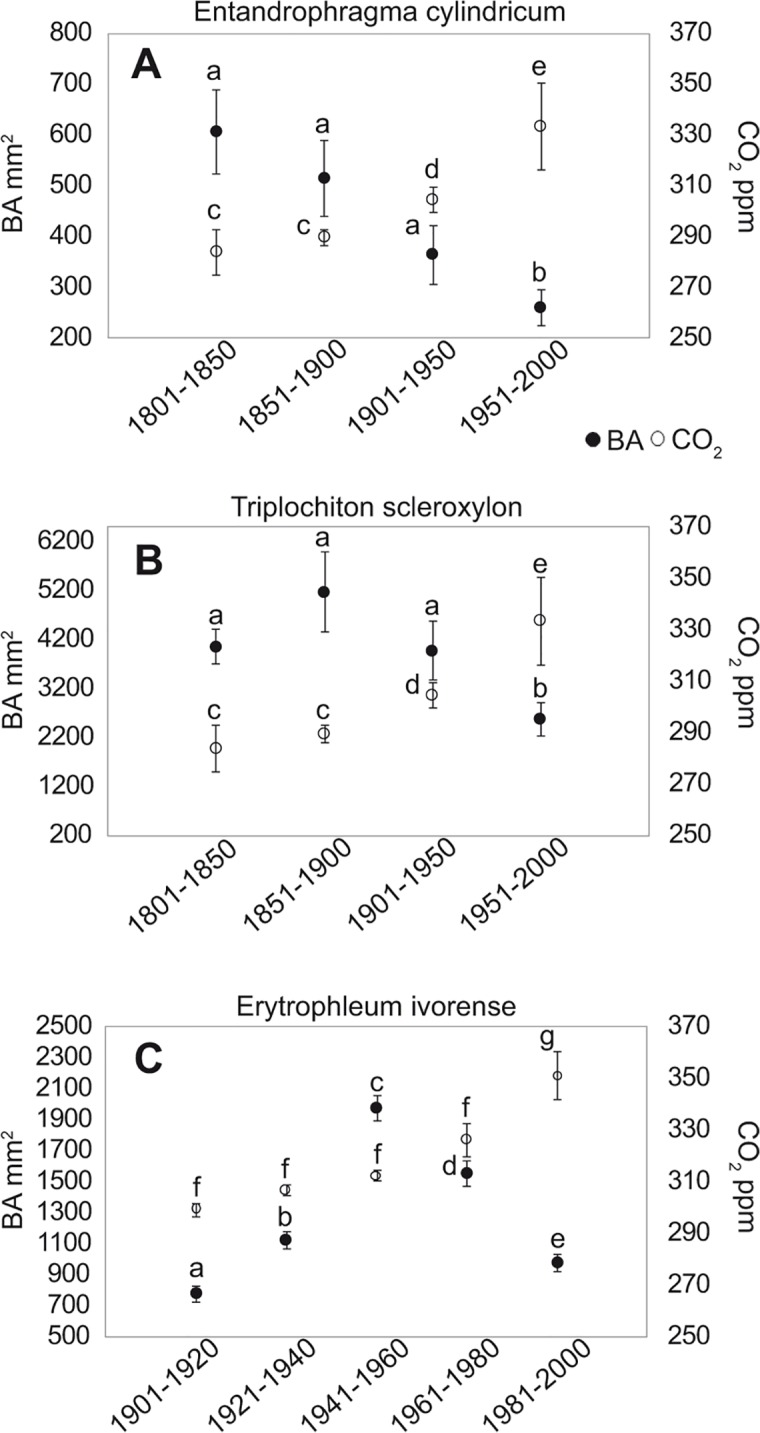
Basal area and CO_2_ concentration data of the three species. BAI (black circle) and CO_2_ concentration (white circle) data grouped into 50-year intervals for *E*. *cylindricum (a)* and *T*. *scleroxylon* (b) for the period 1800–2000. BAI and CO_2_ concentration data grouped into 20-year intervals for *E*. *ivorense* (c) for the period 1900–2000. Different letters correspond to significantly different values for BAI and CO_2_ between different grouped years.

The CO_2_ concentration showed an increasing trend over the two analyzed centuries while the tree growth of the three species did not present any increasing trend in the same period (Fig. 6 and S2–S4 Figs.). Indeed, total tree growth of *E*. *cylindricum*, which is the species with the oldest samples, showed a decreasing trend from 1800 to 2000 in contrast to the CO_2_ increase (Fig. 6a, Fig. 7a, S2 Fig.). *T*. *scleroxylon* showed the same sharp decrease during the twentieth century (Fig. 6b, Fig. 7b, S3 Fig), while the growth trend in the previous 100 years did not show a significant trend (Fig. 7b). Basal area increments of *E*. *ivorense* were summed in 20-year intervals, since the chronology covers only the period 1900–2000 (Fig. 7c). In Fig. 7c (S4 Fig), an increase in growth starting from 1900 to 1941–1960 is evident, followed by a statistically significant decrease during the last 50 years (1961–2000).

**Fig 8:**
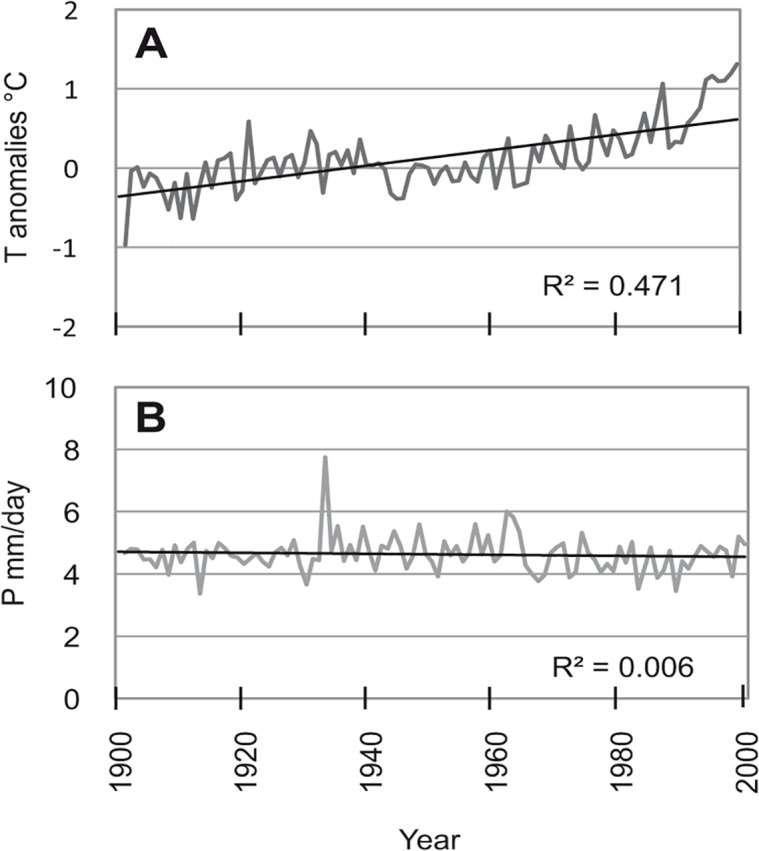
Temperature anomalies and mean annual precipitation. Temperature anomalies (a) and mean annual precipitation (b) for the period 1900–2000. Trend-lines and the corresponding r values are reported (straight black lines.).

On analyzing the climate data for the available time frame (1900–2000), a continuous increase in mean atmospheric temperature can be observed over the whole century, whereas no clear increasing or decreasing trend could be derived for mean annual precipitation. In terms of deviation from the mean value (anomalies), a considerable temperature increase has occurred in recent decades (Fig. 8a), while mean rainfall did not show any clear trend (Fig. 8b).

Multiple regression analysis (Table 2) showed that variations of all species tree growth were negatively correlated with both CO_2_ concentration (r = 0.636 for *E*. *cylindricum*, r = -0.518 for *T*. *scleroxylon* and r = -0.362 for *E*. *ivorense*, *P*<0.0001) and temperature (r = -0.303; r = -0.412; r = -0.313 respectively with *P*<0.0001). There was no evidence for any interaction effect of CO_2_ concentration and temperature on tree growth (*P* = 0.039 for *E*. *cylindricum*, *P* = 0.771 for *T*. *scleroxylon* and *P* = 0.005 for *E*. *ivorense*). No significant relationships were found between BAI of the three species and mean annual precipitation (Table 2).

**Table 2:** Multiple regression analyses of basal area in relation to CO_2_ and climate variables.

Species	Effect	DF	F	P	r
*E*. *cylindricum*	CO_2_	1	54.17	<0.0001	-0.64
	t	1	18.28	<0.0001	-0.30
	p	1	0.15	0.70	-0.07
	CO_2_ * t	1	4.38	0.04	
	CO_2_ * p	1	10.06	0.00	
	t * p	1	0.52	0.47	
*T*. *scleroxylon*	CO_2_	1	51.51	<0.0001	-0.52
	t	1	35.12	<0.0001	-0.41
	p	1	2.09	0.15	-0.02
	CO_2_ * t	1	0.09	0.77	
	CO_2_ * p	1	7.22	0.02	
	t * p	1	1.00	0.32	
*E*. *ivorense*	CO_2_	1	36.77	<0.0001	-0.37
	t	1	35.13	<0.0001	-0.31
	p	1	0.01	0.92	0.00
	CO_2_ * t	1	8.35	0.01	
	CO_2_ * p	1	5.95	0.02	
	t * p	1	1.44	0.23	

Multiple regression analyses of tree basal area (as dependent variable) in relation to CO_2_, temperature, precipitation (independent variables) and their interactions for all three species. DF = degrees of freedom; t = temperature; p = precipitation

## Discussion

Overall data analysis provided evidence that for the three analyzed African species tree growth have decelerated significantly in the last century. The same decelerating trend of tree growth was also reported from permanent forest plots in Panama, Malaysia [59] and Costa Rica [60] and from a recent dendrocronological study across Bolivia, Thailand and Cameroon [42].

The observed decline in tree growth during recent times provides strong evidence against the hypothesis of a continuing rise in tropical tree growth due to elevated resource availability caused by carbon fertilization and/or elevated nutrient deposition [3, 6, 7, 16]. Our findings are in agreement with several published dendrochronological studies where the recent increase in CO_2,_ although it seems to be responsible for a considerable increase of tree water use efficiency, is not followed by an enhancement in tree growth in temperate, boreal and tropical forests [25, 42, 59, 61, 62, 63].

### Potential influence of sampling biases on growth trends

The possible occurrence of sampling biases in the reconstruction of historical growth rate has been recently described [42, 64, 65] and the potential role of such biases needs to be considered in order to minimize the risk of erroneous conclusions [42]. The *big-tree selection bias* is caused by sampling only the biggest trees in a population. As a result, slow-growing small trees are underrepresented in recent times as they did not reach the minimum sample diameter [64]. The *slow-grower survivorship bias* is caused by differences in tree longevity of fast- and slow-growing trees within a population. If fast-growing trees live shorter, they are underrepresented in the ancient portion of the tree ring data set. Both sample biases lead to spurious increases in historical growth rates. However, while the big-tree selection bias leads to growth increases over the most recent time periods, the slow-grower survivorship bias results in growth increases over earlier, historical time periods [64]. To avoid such biases, different sampling strategies have been suggested based on i) selection of trees from smaller size classes ii) fully randomized selection of trees. We included trees of all sizes in our sampling design and we based our sampling approach on a random mechanism, thus minimizing the risk of both sampling biases [42, 64, 65]. A further potential problem that might be encountered in the analysis of long term series of tree rings is posed by age-dependency of the CO_2_ effect on trees [13, 21, 22], i.e. a different radial increment occurring along the life span of the tree due to a higher stimulation of stem biomass growth by CO_2_ increase in the very early stage of tree growth, which becomes less significant in later development stages of the stand [20, 66]. Early tree growth stimulation, often observed in short-term experiments, has been found to gradually decline and disappear after a few years in many experiments [6, 10, 11, 21, 66]. To avoid the confounding age-dependency of CO_2_ effect we used young and old individuals of each species to build the average chronology and we evaluated to what extent detrending methodologies could have influenced our findings (Supporting Information). Since BAI trends for the relatively young trees followed the same pattern as that of the oldest trees, we excluded that the recent growth decline observed in this study can be attributed completely to aging. Indeed, for all the tree species, BAI started to decline before the senescence of each individual, when BAI is naturally expected to decrease [67]. Further, changes in growth of the three different species obtained through the application of the various detrending techniques were similar to those obtained using the raw data. The decrease in growth that characterizes the period under study remains largely unaffected by the application of detrending methods.

### Growth response to CO_2_

Several hypotheses have been suggested in recent publications to explain why the increase in atmospheric CO_2_ concentration would not have a clear positive effect on tree growth. It has been suggested that changes in growth may not be indicative of overall changes in forest productivity [5]. Indeed, it is possible that trees are responding to changing environmental conditions through shifts in their seasonal allocation of photosynthate from stem growth to other pathways, such as root growth, leaf production and/or reproduction [68]. Therefore, total productivity might have remained stable or even increased despite the slowed stem growth, given that tropical trees often experience seasonal cycle growth, possibly with disproportional carbohydrate allocation between the wet and dry season [69]. However, as suggested by Pregitzer et al. (1995) [70], even under this scenario, a decrease in stem growth could have important impacts on carbon sequestration/storage as the residence time of carbon in fine roots, leaves, flowers, or fruits is significantly shorter than in coarse woody tissue.

A second hypothesis is that other environmental limiting factors could override the possible benefits of CO_2_ increase on tree growth [10, 25, 42, 63]. In particular, warmer temperatures could increase plant respiration rates relative to assimilation rates, resulting in a decrease in net carbon assimilation [15, 71]. Moreover, photosynthesis is not linearly related to leaf temperature and the direct effect of warming on CO_2_ assimilation rates depends on how close the tree already is to its thermal optimum. When the latter condition occurs, as often observed in tropical ecosystems, a further increase in temperature might inhibit CO_2_ uptake by pushing the system into supra-optimal temperatures [72], thus reducing photosynthetic rates. This hypothesis is consistent with the rise in local temperature observed at our study site, which tended to be particularly pronounced in the most recent decades of the twentieth century, without a simultaneous increase in rainfall. This would mean that the temperature increase, rather than the atmospheric CO_2_ increase, might have been the dominant driving factor in generating tree net biomass decrease and long-term changes in BAI [25, 59, 63]. In this respect, spatial variation in trend and magnitudes of solar radiation forcing might add to more uniform temperature and CO_2_ concentration variation, with a consequent patchy pattern of tree growth rates [73]. Williams et al. (2007) [74] and Way and Oren (2010) [75] hypothesized that tropical species may be particularly sensitive to ongoing temperature warming since they grow under more constricted ambient temperature ranges than temperate species. Thus they could be more sensitive to climate changes and could prove particularly vulnerable to high temperatures [76]. Indeed, a warmer atmosphere could not only lead to a higher rate of plant respiration and hence a decrease in net primary productivity, but could also induce higher loss of water through evapotranspiration and, hence, conservative strategies aimed at reducing water losses through stomata closure [15], particularly after droughts. This might lead to a lower rate of carbon assimilation and explain the recent tree growth decline reported in this and other studies. Growth-hydraulic limitation trade-offs do exist among tropical trees, and it is possible that fast growing and low wood density species (such as *T*. *scleroxylon* and *E*. *ivorense* to a lesser extent) experience greater increment in wet periods, though being potentially at greater risk of hydraulic stress and consequent reduced growth as soil water availability drops, in comparison with slow growing and high wood density species (such as *E*. *cylindricum*). A species-specific response of trees to climatic and atmospheric conditions is a known phenomenon, reported in other studies. Several examples of physiological adjustments of photosynthetic rates under elevated CO_2_ levels have been reported for some species while no acclimation or even a decline in both conductance and photosynthesis has been observed for others. Species-specific responses were also found in the studies of Laurance et al. (2004) [6] and Lewis et al. (2004) [3], where elevated CO_2_ seemed to have a different effect upon fast-growing pioneer species, with stronger growth enhancement, and upon slower-growing shade-tolerant species, showing a small stimulation effect under elevated CO_2_. This could explain the slightly different behavior of *T*. *scleroxylon* (a light-demander pioneer species) compared to *E*. *cylindricum* (a non-pioneer light-demander with low growth rates). The first could have been affected by the sudden increase in CO_2_ atmospheric level during the beginning of the Industrial Revolution. The second did not show the same trend: growth decline has been evident since the nineteenth century. Additionally, the reported value of mean sensitivity (MS in Table 1) provides information on the strength of dependence on a limiting factor. Thus the high MS for *T*. *scleroxylon* underlines how highly sensitive this species is to the level of available resources and to variations in environmental conditions, including climate.

These results are in agreement with Tandoh et al. (2013) [77], where tree rings of the same three species were analyzed with the ^14^C method and growth simulated using empirical models. They found a lower growth rate in the industrial than in the pre-industrial periods, with differences between *E*. *cylindricum* (which showed lower growth rates) and the other two species. The authors attributed these differences to a species-specific growth rate as well as to an age effect, since only old individuals of *E*. *cylindricum* were analyzed. In our study, the age effect can be considered negligible, because both mature and young trees of each species were measured.

In conclusion, our results indicate that increase in CO_2_ has not stimulated tree growth in the analyzed region of Africa. On the contrary, a growth decline was observed in the last few decades, in accordance with previous studies in the same region [42]. The study did not allow the direct effect of CO_2_ atmospheric concentration and temperature on net tree growth to be disentangled, though climatic regime and, in particular, mean annual temperature were thought to be more relevant than atmospheric CO_2_ concentration in affecting tree growth [78]. The relationship between increased forest productivity and CO_2_ fertilization [8, 9, 79, 80] may not be straightforward, and a whole set of potential limiting factors of tree growth needs to be taken into account to predict how tropical forests will respond to climate change in the coming decades. These uncertainties warrant further tree ring analysis of historical growth trends vs. recent biomass response to natural disturbance patterns to forecast tropical forests productivity, at local and global levels.

## Supporting Information

S1 Fig Running EPS and running RBAR statistics for the three species. Running EPS (black line) and running RBAR statistics (grey line) for (a) *E*. *cylindricum*, (b) *T*. *scleroxylon*, (c) *E*. *ivorense*. EPS and RBAR used 50-year windows, lagged 25 year for *E*. *cylindricum* and *T*. *scleroxylon*, and 20-year windows, lagged 10 year for *E*. *ivorense*. Dashed lines indicate the EPS threshold value of 0.85.
(TIF)

S2 Fig Detrending methods for *E*. *cylindricum*. (a) Mean BAI chronology of *E*. *cylindricum* after 50-year cubic smoothing spline function detrending; (b) Tree growth index (TGI) record obtained through the application of the Regional Curve Standardization technique (TGI RCS) of *E*. *cylindricum*; (c) detrended BAI—dBAI- (black circle) and CO_2_ concentration (white circle) data grouped into 50-year intervals for *E*. *cylindricum* for the period 1800–2000; (d) TGI RCS data (black circle) and CO_2_ concentration (white circle) data grouped into 50-year intervals for *E*. *cylindricum* for the period 1800–2000. Different letters correspond to significantly different values for BAI, TGI RCS and CO_2_ between different grouped years.
(TIF)

S3 Fig Detrending methods for *T*. *scleroxylon*. (a) Mean BAI chronology of *T*. *scleroxylon* after 50-year cubic smoothing spline function detrending; (b) Tree growth index (TGI) record obtained through the application of the Regional Curve Standardization technique (TGI RCS) of *T*. *scleroxylon*; (c) detrended BAI-dBAI- (black circle) and CO_2_ concentration (white circle) data grouped into 50-year intervals for *T*. *scleroxylon* for the period 1800–2000; (d) TGI RCS data (black circle) and CO_2_ concentration (white circle) data grouped into 50-year intervals for *T*. *scleroxylon* for the period 1800–2000. Different letters correspond to significantly different values for BAI, TGI RCS and CO_2_ between different grouped years.
(TIF)

S4 Fig Detrending methods for *E*. *ivorense*. (a) Mean BAI chronology of *E*. *ivorense* after 20-year cubic smoothing spline function detrending; (b) Tree growth index (TGI) record obtained through the application of the Regional Curve Standardization technique (TGI RCS) of *E*. *ivorense*; (c) detrended BAI-dBAI- (black circle) and CO_2_ concentration (white circle) data grouped into 20-year intervals for *E*. *ivorense* for the period 1900–2000; (d) TGI RCS data (black circle) and CO_2_ concentration (white circle) data grouped into 20-year intervals for *E*. *ivorense* for the period 1900–2000. Different letters correspond to significantly different values for BAI, TGI RCS and CO_2_ between different grouped years.
(TIF)

S5 Fig Scatter-plots for *E*. *cylindricum*. (a) Scatter-plot of original BAI versus detrended BAI series (dBAI) of *E*. *cylindricum*; (b) Scatter-plot of original BAI versus TGI RCS series of *E*. *cylindricum*; (c) Scatter-plot of original BAI versus detrended tree-ring width series of *E*. *cylindricum*; (d) Scatter-plot TGI RCS versus detrended BAI series of *E*. *cylindricum*.
(TIF)

S6 Fig Scatter-plots for *T*. *scleroxylon*. (a) Scatter-plot of original BAI versus detrended BAI series (dBAI) of *T*. *scleroxylon*; (b) Scatter-plot of original BAI versus TGI RCS series of *T*. *scleroxylon*; (c) Scatter-plot of original BAI versus detrended tree-ring width series of *T*. *scleroxylon*; (d) Scatter-plot TGI RCS versus detrended BAI series of *T*. *scleroxylon*.
(TIF)

S7 Fig Scatter-plots for *E*. *ivorense*. (a) Scatter-plot of original BAI versus detrended BAI series (dBAI) of *E*. *ivorense*; (b) Scatter-plot of original BAI versus TGI RCS series of *E*. *ivorense*; (c) Scatter-plot of original BAI versus detrended tree-ring width series of *E*. *ivorense*; (d) Scatter-plot TGI RCS versus detrended BAI series of *E*. *ivorense*.
(TIF)

S1 Information Sensitivity analysis to the detrending method. Since diameter growth of trees changes with tree size (and age), it is important to separate ontogenetic growth changes from potential growth changes over time. Therein we applied a sensitivity analysis to demonstrate that our results are not influenced by the used approach. BAI curves were detrended with a 50-year cubic smoothing spline function, while the tree-ring width chronologies were detrended with the regional curve standardization (RCS) technique and the final results were compared to those presented in the original manuscript (where Sapele ring-width series were detrended with a 50-year cubic smoothing spline function, the shorter Ayous and Tali series with 20-years function). The detrended BAI chronologies were obtained using cubic smoothing spline curves that are efficient to remove non-climatic noise, such as long-term trends and effects of localized disturbance events that characterize natural forest dynamics; at the same time they can cause the removal of possible low-frequency climatic information (S2a, S4a, S5a Figs). The RCS approach has the potential to preserve the evidence of long-time scale forcing of tree growth: the measurement series were aligned by cambial age, scaled using the power-transformation method, and the arithmetic mean of ring width for each ring age was calculated. A regional curve (RC) was then created by applying a flexible smoothing (Hugershoff) to the age series of arithmetic means. Next, each one of the original ring-width measurement series was divided by the RC value for the appropriate ring age to create standardized series. Finally, the standardized series were realigned by calendar year and averaged using a bi-weight robust mean to create the tree growth index (TGI RCS- S2b, S3b, S4b Figs). As shown by S2c, d Figs.; S3c, d Figs.; S4c, d Figs. tree growth of the three species present a decreasing trend in the last decades in comparison with the CO_2_ increasing trend. The changes in growth over time thus appear to be adequately recovered. In addition, as shown by the scatter-plots of S5, S6, S7 Figs., the standardization methods yielded very similar changes in mean species growth for the study period.
(DOCX)
