# Correction: Long Tree-Ring Chronologies Provide Evidence of Recent Tree Growth Decrease in a Central African Tropical Forest

**DOI:** 10.1371/journal.pone.0126168

**Published:** 2015-04-13

**Authors:** The PLOS ONE Staff

The fifth author’s name is spelled incorrectly. The correct name is: Roberto Cazzolla Gatti. The correct citation is: Battipaglia G, Zalloni E, Castaldi S, Marzaioli F, Cazzolla Gatti R, et al. (2015) Long Tree-Ring Chronologies Provide Evidence of Recent Tree Growth Decrease in a Central African Tropical Forest. PLoS ONE 10(3): e0120962. doi:10.1371/journal.pone.0120962
