# Supplementary figures and images for: Long Tree-Ring Chronologies Provide Evidence of Recent Tree Growth Decrease in a Central African Tropical Forest
Source: PLoS One. 2015 Mar 25;10(3):e0120962. doi: 10.1371/journal.pone.0120962 (PMC4373839; doi:10.1371/journal.pone.0120962)

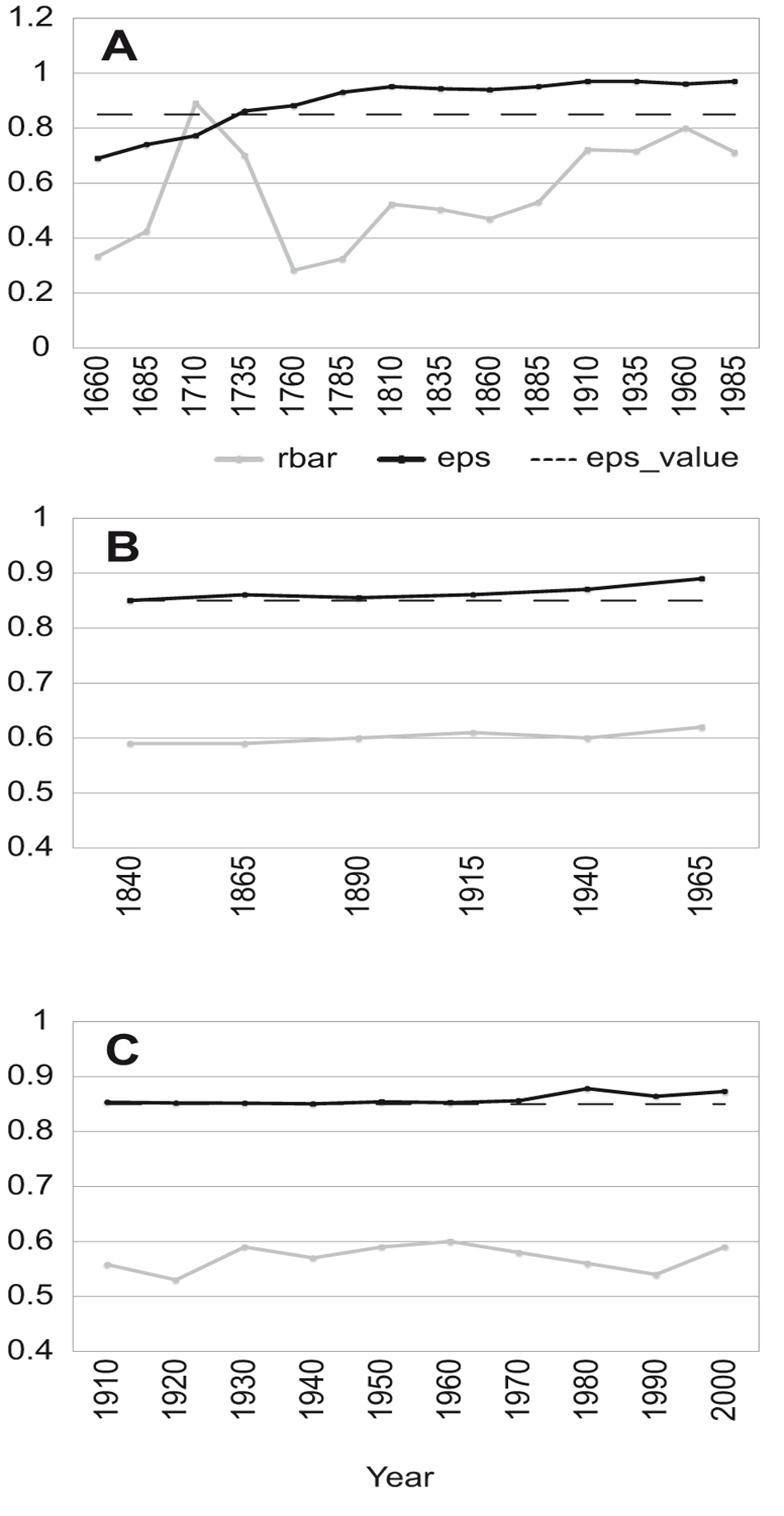

Supplement: S1 Fig — Running EPS (black line) and running RBAR statistics (grey line) for (a) E. cylindricum, (b) T. scleroxylon, (c) E. ivorense. EPS and RBAR used 50-year windows, lagged 25 year for E. cylindricum and T. scleroxylon, and 20-year windows, lagged 10 year for E. ivorense. Dashed lines indicate the EPS threshold value of 0.85. (TIF) [file pone.0120962.s001.tif]

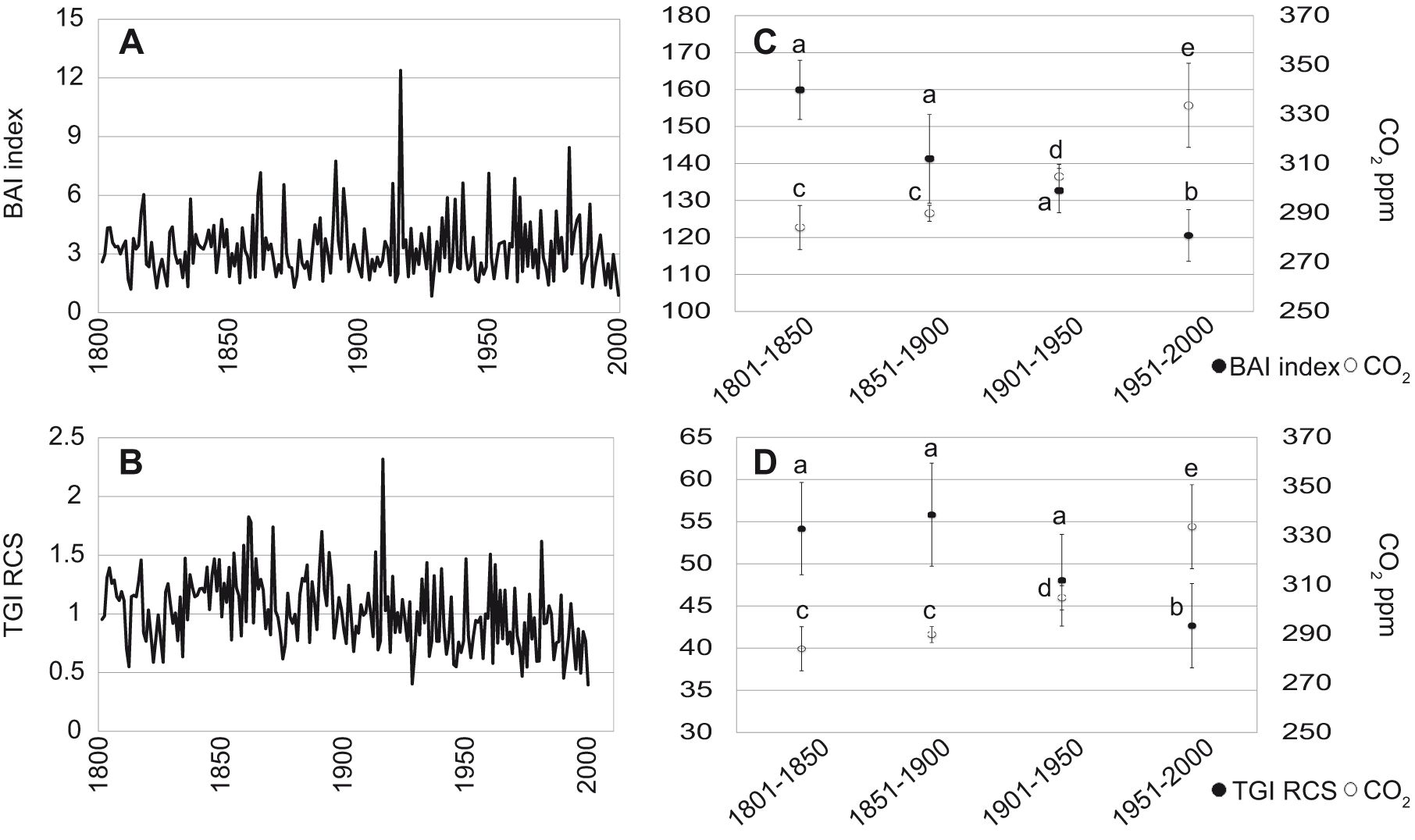

Supplement: S2 Fig — (a) Mean BAI chronology of E. cylindricum after 50-year cubic smoothing spline function detrending; (b) Tree growth index (TGI) record obtained through the application of the Regional Curve Standardization technique (TGI RCS) of E. cylindricum; (c) detrended BAI—dBAI- (black circle) and CO2 concentration (white circle) data grouped into 50-year intervals for E. cylindricum for the period 1800–2000; (d) TGI RCS data (black circle) and CO2 concentration (white circle) data grouped into 50-year intervals for E. cylindricum for the period 1800–2000. Different letters correspond to significantly different values for BAI, TGI RCS and CO2 between different grouped years. (TIF) [file pone.0120962.s002.tif]

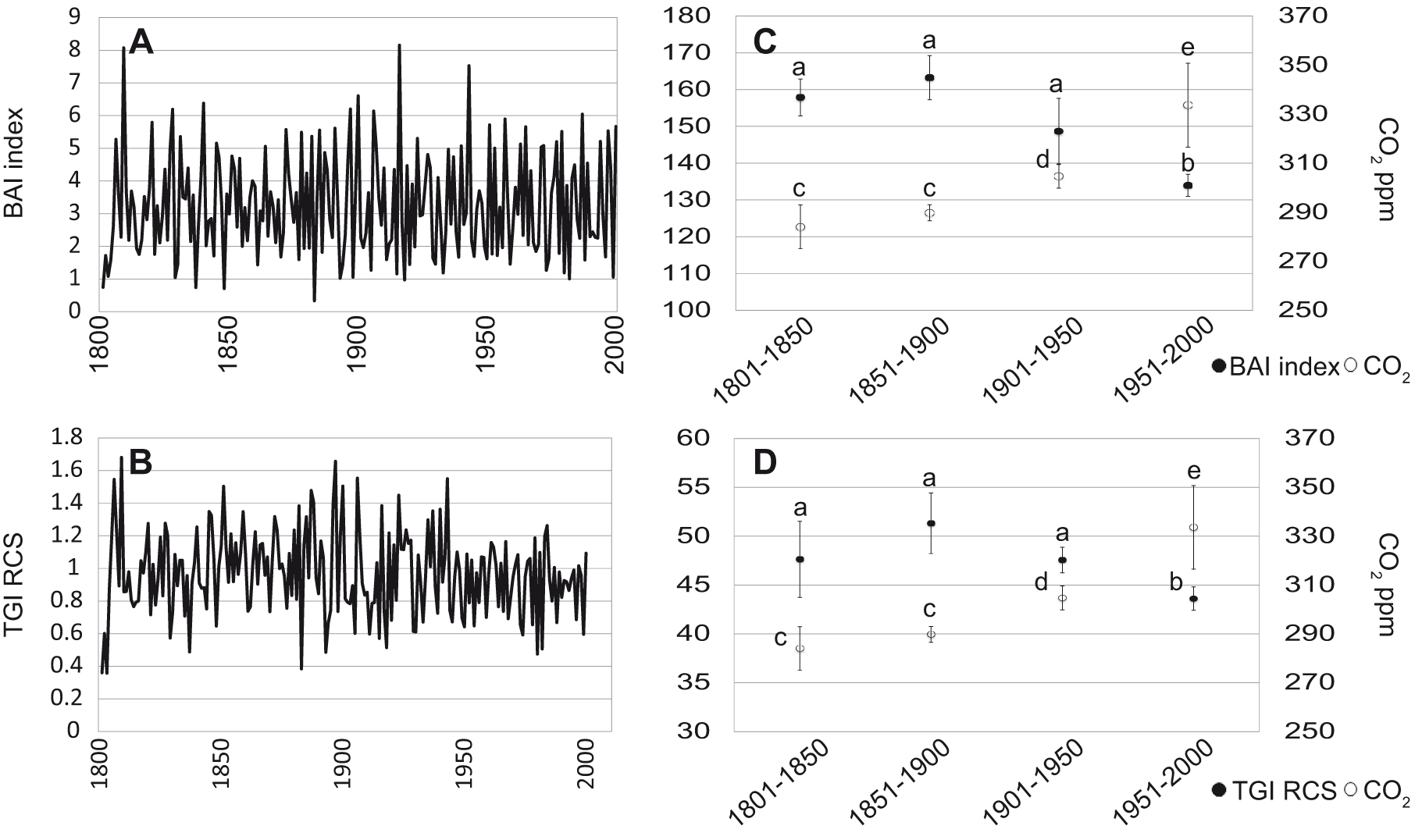

Supplement: S3 Fig — (a) Mean BAI chronology of T. scleroxylon after 50-year cubic smoothing spline function detrending; (b) Tree growth index (TGI) record obtained through the application of the Regional Curve Standardization technique (TGI RCS) of T. scleroxylon; (c) detrended BAI-dBAI- (black circle) and CO2 concentration (white circle) data grouped into 50-year intervals for T. scleroxylon for the period 1800–2000; (d) TGI RCS data (black circle) and CO2 concentration (white circle) data grouped into 50-year intervals for T. scleroxylon for the period 1800–2000. Different letters correspond to significantly different values for BAI, TGI RCS and CO2 between different grouped years. (TIF) [file pone.0120962.s003.tif]

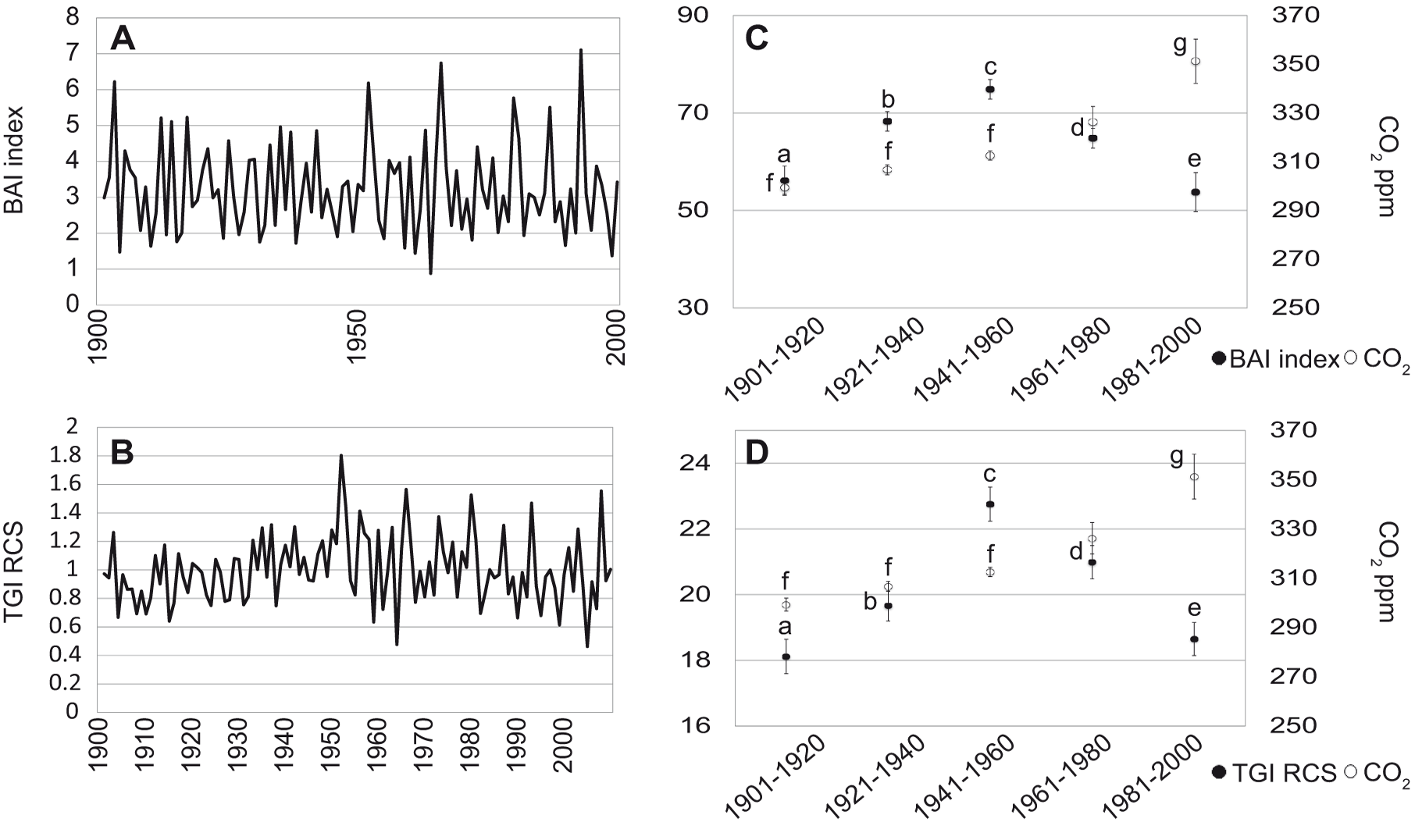

Supplement: S4 Fig — (a) Mean BAI chronology of E. ivorense after 20-year cubic smoothing spline function detrending; (b) Tree growth index (TGI) record obtained through the application of the Regional Curve Standardization technique (TGI RCS) of E. ivorense; (c) detrended BAI-dBAI- (black circle) and CO2 concentration (white circle) data grouped into 20-year intervals for E. ivorense for the period 1900–2000; (d) TGI RCS data (black circle) and CO2 concentration (white circle) data grouped into 20-year intervals for E. ivorense for the period 1900–2000. Different letters correspond to significantly different values for BAI, TGI RCS and CO2 between different grouped years. (TIF) [file pone.0120962.s004.tif]

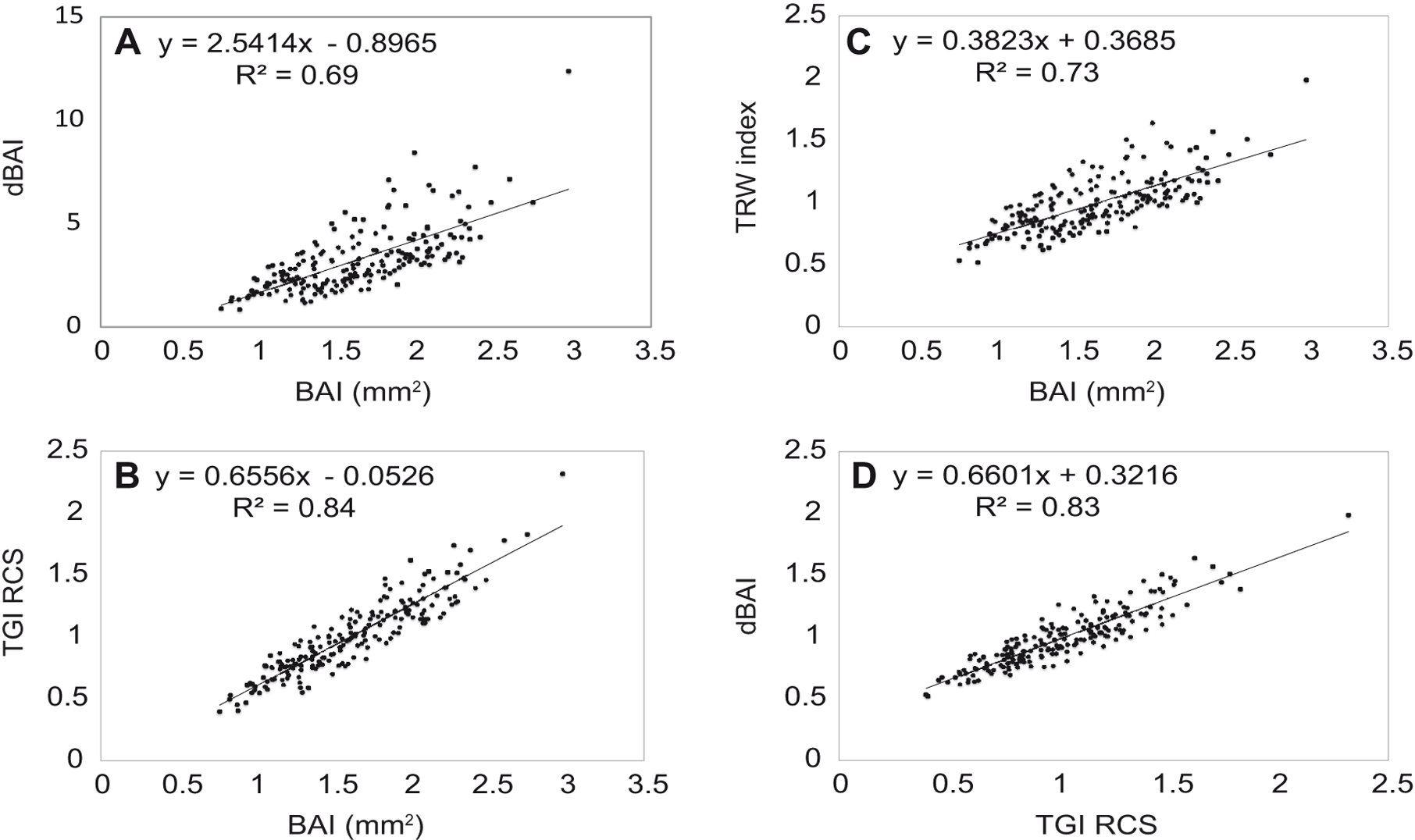

Supplement: S5 Fig — (a) Scatter-plot of original BAI versus detrended BAI series (dBAI) of E. cylindricum; (b) Scatter-plot of original BAI versus TGI RCS series of E. cylindricum; (c) Scatter-plot of original BAI versus detrended tree-ring width series of E. cylindricum; (d) Scatter-plot TGI RCS versus detrended BAI series of E. cylindricum. (TIF) [file pone.0120962.s005.tif]

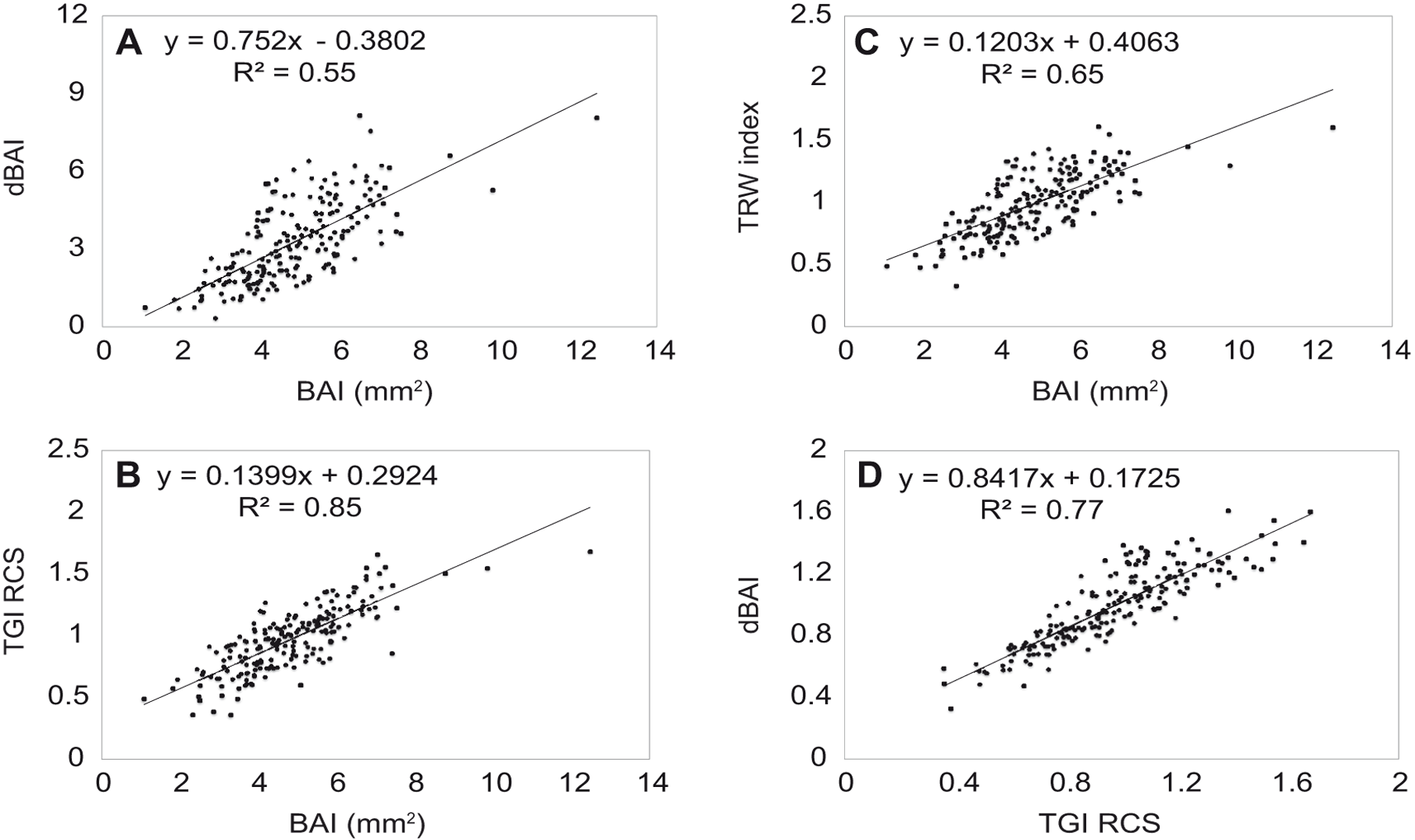

Supplement: S6 Fig — (a) Scatter-plot of original BAI versus detrended BAI series (dBAI) of T. scleroxylon; (b) Scatter-plot of original BAI versus TGI RCS series of T. scleroxylon; (c) Scatter-plot of original BAI versus detrended tree-ring width series of T. scleroxylon; (d) Scatter-plot TGI RCS versus detrended BAI series of T. scleroxylon. (TIF) [file pone.0120962.s006.tif]

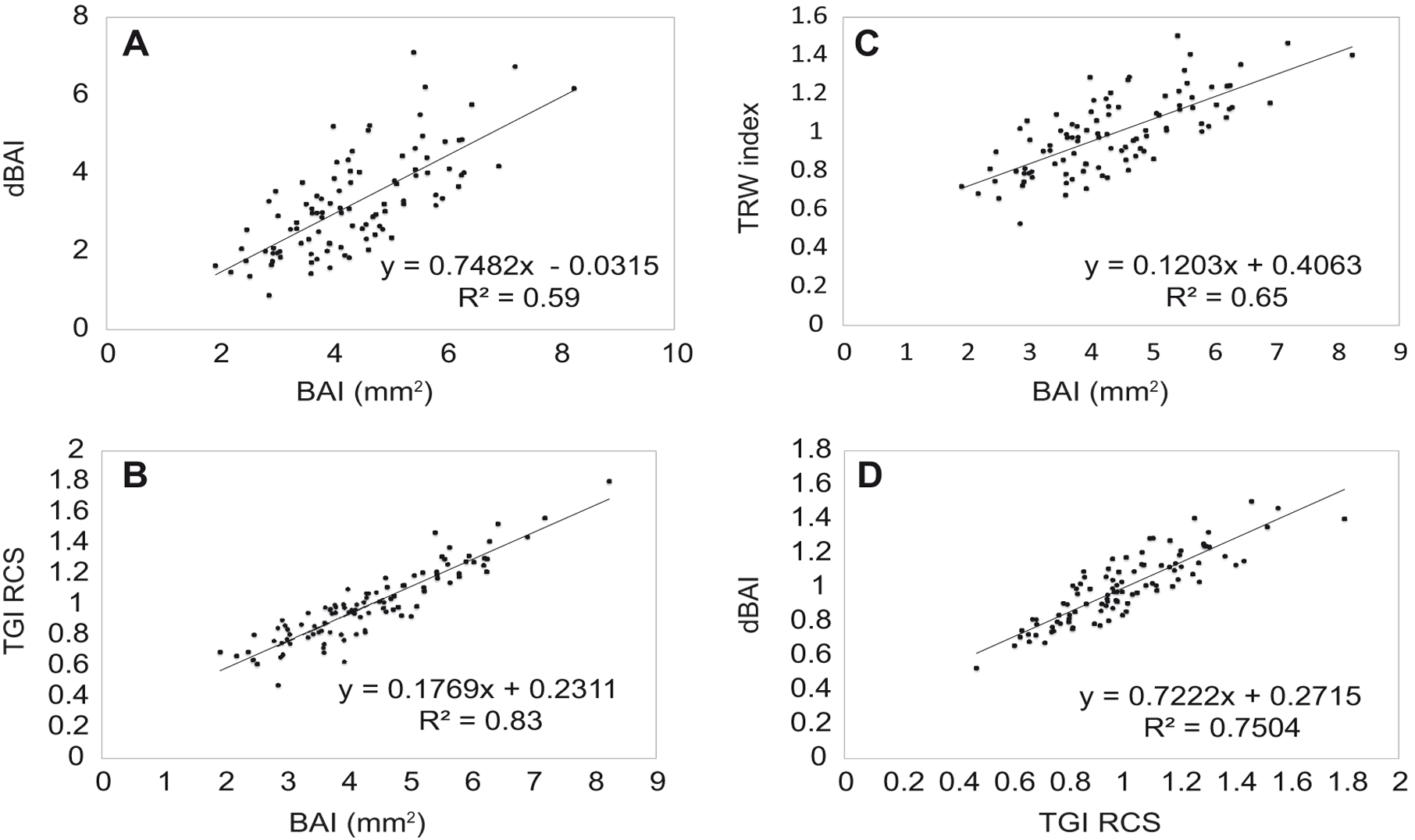

Supplement: S7 Fig — (a) Scatter-plot of original BAI versus detrended BAI series (dBAI) of E. ivorense; (b) Scatter-plot of original BAI versus TGI RCS series of E. ivorense; (c) Scatter-plot of original BAI versus detrended tree-ring width series of E. ivorense; (d) Scatter-plot TGI RCS versus detrended BAI series of E. ivorense. (TIF) [file pone.0120962.s007.tif]
