# Supplementary material for: Long Tree-Ring Chronologies Provide Evidence of Recent Tree Growth Decrease in a Central African Tropical Forest
Source: PLoS One. 2015 Mar 25;10(3):e0120962. doi: 10.1371/journal.pone.0120962 (PMC4373839; doi:10.1371/journal.pone.0120962)
Supplement: S1 Information — Since diameter growth of trees changes with tree size (and age), it is important to separate ontogenetic growth changes from potential growth changes over time. Therein we applied a sensitivity analysis to demonstrate that our results are not influenced by the used approach. BAI curves were detrended with a 50-year cubic smoothing spline function, while the tree-ring width chronologies were detrended with the regional curve standardization (RCS) technique and the final results were compared to those presented in the original manuscript (where Sapele ring-width series were detrended with a 50-year cubic smoothing spline function, the shorter Ayous and Tali series with 20-years function). The detrended BAI chronologies were obtained using cubic smoothing spline curves that are efficient to remove non-climatic noise, such as long-term trends and effects of localized disturbance events that characterize natural forest dynamics; at the same time they can cause the removal of possible low-frequency climatic information (S2a, S4a, S5a Figs). The RCS approach has the potential to preserve the evidence of long-time scale forcing of tree growth: the measurement series were aligned by cambial age, scaled using the power-transformation method, and the arithmetic mean of ring width for each ring age was calculated. A regional curve (RC) was then created by applying a flexible smoothing (Hugershoff) to the age series of arithmetic means. Next, each one of the original ring-width measurement series was divided by the RC value for the appropriate ring age to create standardized series. Finally, the standardized series were realigned by calendar year and averaged using a bi-weight robust mean to create the tree growth index (TGI RCS- S2b, S3b, S4b Figs). As shown by S2c, d Figs.; S3c, d Figs.; S4c, d Figs. tree growth of the three species present a decreasing trend in the last decades in comparison with the CO2 increasing trend. The changes in growth over time thus appear to be adeq [file pone.0120962.s008.docx]

**S1 information.** **Sensitivity analysis to the detrending method**

Since diameter growth of trees changes with tree size (and age), it is important to separate ontogenetic growth changes from potential growth changes over time. Therein we applied a sensitivity analysis to demonstrate that our results are not influenced by the used approach.

BAI curves were detrended with a 50-year cubic smoothing spline function, while the tree-ring width chronologies were detrended with the regional curve standardization (RCS) technique and the final results were compared to those presented in the original manuscript (where Sapele ring-width series were detrended with a 50-year cubic smoothing spline function, the shorter Ayous and Tali series with 20-years function).

The detrended BAI chronologies were obtained using cubic smoothing spline curves that are efficient to remove non-climatic noise, such as long-term trends and effects of localized disturbance events that characterize natural forest dynamics; at the same time they can cause the removal of possible low-frequency climatic information (S2a, S4a, S5a Figs)

The RCS approach has the potential to preserve the evidence of long-time scale forcing of tree growth: the measurement series were aligned by cambial age, scaled using the power-transformation method, and the arithmetic mean of ring width for each ring age was calculated. A regional curve (RC) was then created by applying a flexible smoothing (Hugershoff) to the age series of arithmetic means. Next, each one of the original ring-width measurement series was divided by the RC value for the appropriate ring age to create standardized series. Finally, the standardized series were realigned by calendar year and averaged using a bi-weight robust mean to create the tree growth index (TGI RCS- S2b, S3b, S4b figs). As shown by S2c,d; S3c,d; S4c,d Figs tree growth of the three species present a decreasing trend in the last decades in comparison with the CO_2_ increasing trend. The changes in growth over time thus appear to be adequately recovered. In addition, as shown by the scatter-plots of S5, S6, S7 Figs, the standardization methods yielded very similar changes in mean species growth for the study period.
